# Epidemiological aspects of HIV infection and AIDS among indigenous populations

**DOI:** 10.11606/S1518-8787.2019053000362

**Published:** 2019-09-02

**Authors:** Samara Vilas-Bôas Graeff, Renata Palópoli Pícolli, Rui Arantes, Vivianne de Oliveira Landgraf de Castro, Rivaldo Venâncio da Cunha

**Affiliations:** I Prefeitura Municipal de Campo Grande. Secretaria Municipal de Saúde. Campo Grande, MS, Brasil; II Fundação Oswaldo Cruz de Mato Grosso do Sul. Campo Grande, MS, Brasil; III Fundação Oswaldo Cruz. Escola Nacional de Saúde Pública Sérgio Arouca. Departamento de Endemias. Rio de Janeiro, RJ, Brasil; IV Universidade Federal de Mato Grosso do Sul. Laboratório de Imunologia Clínica. Faculdade de Ciências Farmacêuticas, Alimentos e Nutrição. Campo Grande, MS, Brasil; V Universidade Federal de Mato Grosso do Sul. Faculdade de Medicina. Campo Grande, MS, Brasil

**Keywords:** Health of Indigenous Peoples, HIV Infections, epidemiology, Acquired Immunodeficiency Syndrome, epidemiology, AIDS HIV Seroprevalence, trends, Serodiagnosis, trends

## Abstract

**OBJECTIVE:**

To describe the epidemiological aspects of HIV infection and AIDS among indigenous peoples of the state of Mato Grosso do Sul, Brazil.

**METHODS:**

This is a descriptive epidemiological study on the occurrence and distribution of HIV infection and AIDS in the indigenous population assisted by the *Distrito Sanitário Especial Indígena* (Indigenous Special Health District) Mato Grosso do Sul between 2001 and 2014, based on three secondary databases. Annual rates of HIV and AIDS detection and prevalence were calculated, considering case distribution according to village, Health Base Pole and sociodemographic variables. Accumulated rates of detection, mortality and case fatality were calculated by ethnic group and for the Health Base Pole with the highest number of cases.

**RESULTS:**

The HIV detection rate fluctuated between 0.0 and 18.0/100 thousand people in the study period. For AIDS, there was no notification before 2007, but in 2012 its rate reached 16.6/100 thousand. HIV prevalence grew between 2001 and 2011, and it continuously grew for AIDS starting from 2007. The highest HIV detection rates occurred among Guarani peoples (167.1/100 thousand) and for AIDS, among the Kaiowá peoples (79.3/100 thousand); mortality and fatality rates were higher among the Kaiowá. Regarding the Dourados Health Base Pole, the AIDS detection rate increased, and the mortality and fatality rates decreased.

**CONCLUSIONS:**

HIV infection and AIDS have been increasing among indigenous peoples, with distribution of the disease mainly in the Health Base Poles of the southern region of the state, where greater economic and social vulnerability are also observed. The endemic character of HIV and AIDS can become epidemic in some years given the existence of cases in other villages in the state. Its occurrence among the Guarani and Kaiowá populations indicates the need for expanded diagnosis, access to treatment and prevention measures.

## INTRODUCTION

Acquired immunodeficiency syndrome (AIDS) has already affected millions of people worldwide. By 2015, about 36.7 million people were living with the virus^a^ . In Brazil, from 1980 to June 2017, 882,810 cases of AIDS were recorded^b^ . Between 2006 and 2016, there was a trend towards the stabilization of the AIDS detection rate, considering a 18.5/100 thousand inhabitants mean in 2016. However, in the state of Mato Grosso do Sul, an 8.2% growth occurred in the period, represented by a 19.8/100 thousand inhabitants rate in 2016, the highest in the Midwest region^b^ .

The impact of AIDS among indigenous populations is still little known. However, studies have highlighted the greater vulnerability of these population groups to HIV transmission, related to bad living conditions, lower socioeconomic and educational level, social exclusion and difficulty in accessing health services^1 , 2^ . Information from countries such as the United States, Canada, New Zealand and Australia indicates a significant increase in the HIV detection rate among indigenous peoples in recent decades^3 - 5^ . In general, this rate is higher among indigenous individuals than non-indigenous, indicating health inequities in the access to diagnosis and treatment of the disease^1 , 2 , 5^ .

The first case of AIDS in indigenous populations in Brazil was recorded in 1987, and only 33 cases were known until 1999. This number increased to 1,042 between 2000 and June 2017^b^ . However, the impact of AIDS on indigenous peoples cannot be characterized by absolute numbers and/or rates only. The sociocultural, economic, demographic, and geographic dimensions of each indigenous people, associated with the process of interaction with non-indigenous society, expose them to unique risk factors that lead to greater vulnerability when compared with other Brazilian population groups^1 , 6 ,c^.

The provision of health services for indigenous peoples also has repercussions in this scenario. In recent Brazilian history, the health care of indigenous peoples gained strength in 1999 with the creation of the *Subsistema de Atenção à Saúde Indígena* , within the scope of the Unified Health System (Indigenous Health Care Subsystem – Sasi-SUS)^d^ .

Considering the need for investigations that fill the gaps related to the occurrence of HIV and AIDS among indigenous peoples, and that can subsidize the formulation of appropriate care policies directed to this population, this study aimed to describe the epidemiological aspects of these cases among the indigenous peoples of the state of Mato Grosso do Sul, from 2001 to 2014.

## METHODS

This is a descriptive study on the occurrence and distribution of HIV and AIDS cases on the indigenous population of the state of Mato Grosso do Sul, assisted by the *Distrito Sanitário Especial Indígena Mato Grosso do Sul* (Indigenous Special Health District – DSEI-MS), from 2001 to 2014. Mato Grosso do Sul has the second largest indigenous population in Brazil, with 73,295 self-declared individuals^e^ . The DSEI-MS has 14 Health Base Poles, which are the first reference for the multidisciplinary teams of indigenous health (EMSI) that operate in the villages and care for 70,032 people from eight ethnic groups (Kaiowá, Guarani Ñandeva, Terena, Kadiwéu, Kinikinau, Guató, Ofaié and Atikun) distributed in 75 villages and 26 camps, located in 31 of the 79 municipalities of the state^f^ .

This study comprised individuals with diagnoses of HIV infection from the records of daily evaluations made by the EMSI, and of AIDS from the *Sistema de Informação de Agravos de Notificação* (Information System of Notifiable Diseases – Sinan), being selected by the indigenous race/color variable, who lived in villages and camps of the state. The exclusion criteria were duplicate cases, indigenous people living in villages/urban areas not assisted by DSEI-MS, and children exposed to HIV during pregnancy.

The data recorded by the EMSI during the study period were obtained from June 2014 to January 2015 by manually linking the name, date of birth and name of the mother to three databases: 1. records from daily evaluations made by EMSI; 2. the Sinan database; 3. *Sistema de Informação de Atenção à Saúde Indígena* (Indigenous Health Care Information System – Siasi). The place of residence was considered as the one registered at the time of the HIV diagnosis by the EMSI or the submitted to Sinan for AIDS cases. Sociodemographic variables (ethnic group, sex, date of birth and schooling) were obtained from Siasi, as well as the population bases used for calculating the rates described below.

The number of newly recorded cases of each was used to calculate the annual rates of HIV and AIDS detection. People directly diagnosed with AIDS were only considered for the AIDS detection rate. To include people diagnosed in areas unassisted by DSEI-MS, the territory to which the person moved to was considered, as well as the year in which it occurred.

The number of old and new HIV cases of each year was used to calculate HIV prevalence. Deaths, cases that evolved to AIDS, and people who moved from the areas served by DSEI-MS were excluded. The number of old cases and the new diagnoses was used to calculate AIDS prevalence, in addition to those that evolved to AIDS in that year. Deaths and people who moved from the areas served by DSEI-MS were excluded.

The analysis of the association between the years and the detection rate of HIV and AIDS, as well as the prevalence, was performed using Pearson’s chi-square test with Bonferroni correction when necessary, using the IBM SPSS^®^ statistical program, version 24.0, considering a 5% significance level.

The other results were presented as descriptive statistics. The spatial distribution of HIV and AIDS cases in the area covered by DSEI-MS was determined according to the place of residence (village and Health Base Pole) at the time of diagnosis. HIV and AIDS detection rates, as well as accumulated AIDS mortality and fatality for the whole period were estimated, considering the total population of each ethnic group in 2014. The accumulated rates were also calculated for the three Health Base Poles that concentrated the most diagnoses, divided in two periods (from 2001 to 2007, considering the 2007 base population) and (from 2008 to 2014, considering the 2014 base population).

This study was approved by the *Comissão Nacional de Ética em Pesquisa* (Brazilian Research Ethics Commission – CONEP) under Opinion 707.439/2014.

## RESULTS

HIV infection detection rates showed annual fluctuations in the study period, with values ranging from zero to 18.0/100 thousand people. The highest rates were recorded in 2006 (17.8/100 thousand) and 2011 (18.0/100 thousand). Regarding AIDS cases, there were no records of the disease between 2001 and 2006. From 2007, an increase was observed until 2012, when the detection rate reached 16.6/100 thousand. In 2012 and 2013, AIDS detection rates were higher than HIV detection rates ( Figure ).

**Figure:**
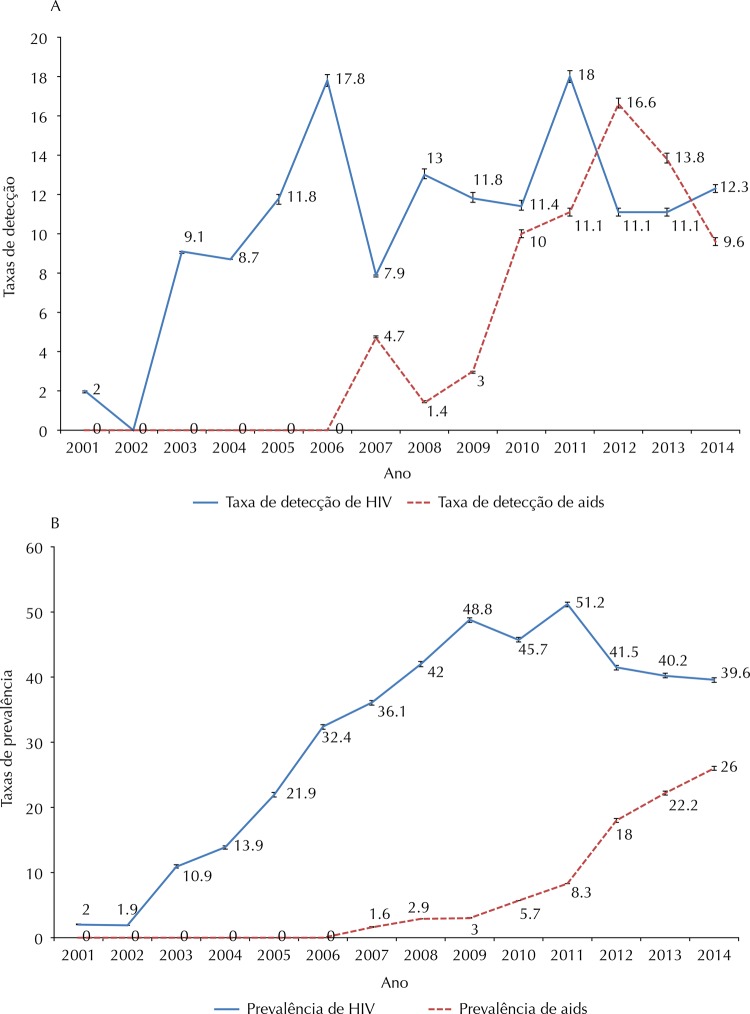
Annual rates (per 100 thousand inhabitants) of detection (A) and prevalence (B), and the respective confidence intervals (95%CI) of HIV infection and AIDS cases in the indigenous population served by the Indigenous Special Health District Mato Grosso do Sul. Mato Grosso do Sul, Brazil, 2001–2014.

Annual HIV prevalence rates increased in the period from 2001 to 2011, and decreased from 2011 onward, decreasing from 51.2/100 thousand to 39.6/100 thousand in 2014. For AIDS, the prevalence rate increased continuously starting from 2007, gradually approaching the HIV infection prevalence after 2012 ( Figure ).

During the study period, 97 HIV infection cases and 50 AIDS cases were recorded, involving 103 people, of whom 6 were directly diagnosed with AIDS. The distribution of cases was quite uneven despite HIV being found in 80% of the Health Base Poles and AIDS in 64% of them. Jointly, the Centers of Dourados, Amambai and Iguatemi concentrated 69% of HIV infection cases and 68% AIDS cases. The Dourados Health Base Pole alone accounted for 34% of HIV infection cases. The Jaguapirú, Amambai, Taquapery and Porto Lindo villages recorded 52% of the AIDS cases, 20% of which were in the Jaguapiru village (Dourados Health Base Pole) ( Table 1 ).

**Table 1:** Distribution HIV infection and AIDS cases at the time of diagnosis, by Health Base Pole and village, and accumulated detection rate (per 100 thousand inhabitants), by Health Base Pole, considering the 2014 base population. Indigenous Special Health District Mato Grosso do Sul. Mato Grosso do Sul, Brazil, 2001–2014.

Health Base Pole	Village (2014 population)	HIV			AIDS		

		Village	Health Base Pole	Det rt HD	Village	Health Base Pole	Det rt HD

		n (%)	n (%)	/100 thousand	n (%)	n (%)	/100 thousand
Dourados	Bororó (6,341)	16 (16.5)	33 (34.0)	217.3	4 (8.0)	14 (28.0)	92.2
	Jaguapirú (6,753)	17 (17.5)			10 (20.0)		
Amambai	Amambai (7,031)	12 (12.4)	23 (23.7)	177.6	6 (12.0)	15 (30.0)	115.8
	Limão Verde (1,602)	4* (4.1)			4 (8.0)		
	Taquapery (3,089)	7 (7.2)			5 (10.0)		
Iguatemi	Porto Lindo (3,946)	11* (11.3)	11 (11.3)	215.4	5 (10.0)	5 (10.5)	97.9
Aquidauana	Aldeinha (398)	1 (1.0)	7 (7.2)	103.4	0 (0.0)	3 (6.0)	44.3
	Bananal (1,109)	3 (3.1)			2 (4.0)		
	Limão Verde (1,161)	2* (2.1)			0 (0.0)		
	Ypegue (736)	1 (1.0)			1 (2.0)		
Caarapó	Caarapó (4,694)	5 (5.2)	6 (6.2)	95.4	4 (8.0)	4 (8.0)	63.6
	Taquara (283)	1 (1.0)			0 (0.0)		
Miranda	Moreira (1,123)	1 (1.0)	5 (5.2)	68.0	1 (2.0)	3 (6.0)	40.8
	Passarinho (1,221)	4 (4.1)			2 (4.0)		
Paranhos	Pirajuí (2,219)	3 (3.1)	3 (3.1)	60.9	1 (2.0)	1 (2.0)	20.3
Sidrolândia	Buriti (953)	2 (2.1)	3 (3.1)	67.6	1 (2.0)	2 (4.0)	45.1
	Córrego do Meio (574)	1 (1.0)			1 (2.0)		
Tacuru	Sassoró (2,111)	3* (3.1)	3 (3.1)	99.2	3* (6.0)	3 (6.0)	99.2
Brasilândia	Ofaié (105)	2 (2.1)	2 (2.1)	1904.8	0 (0.0)	0 (0.0)	0.0
Bodoquena	Alves de Barros (839)	1 (1.0)	1 (1.0)	103.3	0 (0.0)	0 (0.0)	0.0

Total		97 (100.0)	97 (100.0)	132.5	50 (100.0)	50 (100.0)	68.3

Det rt HD: detection rate by Health Base Pole

*One (1) case diagnosed in an individual who resided in a municipality at the time of diagnosis and who then moved to the village during the study period was added.

Regarding the accumulated detection rates of HIV and AIDS by Health Base Pole, there were some ordinal differences in distribution. The Brasilândia Health Base Pole had the highest HIV detection rate (1,904.8/100 thousand), but its population size must be considered. The following Health Base Poles were Dourados (217.3/100 thousand), Iguatemi (215.4/100 thousand) and Amambai (177.6/100 thousand). For the AIDS cases, the detection rate was higher for the Health Base Pole of Amambai (115.8/100 thousand), Tacuru (99.2/100 thousand) and Iguatemi (97.9/100 thousand), leaving the Dourados Base out of the first three places ( Table 1 ).

The age group with the highest incidence of HIV and AIDS infection was 20 to 39 years old, with 43 (44.3%) and 22 (44.0%) cases, respectively. There was statistical significance for HIV cases, for a 0.040 p-value. In the distribution by sex, most HIV and AIDS cases occurred among women, corresponding to 60 (61.9%) and 30 (60%) cases, respectively. We note that HIV infection occurred earlier in women than in men, given that in the 10-19 age group the ratio between the sexes was approximately nine women for each man ( Table 2 ).

**Table 2:** Distribution of HIV infection and AIDS cases according to sociodemographic variables. Indigenous Special Health District Mato Grosso do Sul. Mato Grosso do Sul, Brazil, 2001–2014.

Variable	HIV				AIDS			
	
	Sex			p	Sex			p
	
	F	M	Total		F	M	Total	
					
	n (%)	n (%)	n (%)		n (%)	n (%)	n (%)	
Age group (years)				0.040				0.536
0–9	1 (1.7)	0 (0.0)	1 (1.0)		0 (0.0)	0 (0.0)	0 (0.0)	
10–19	17 (28.3)	2 (5.4)	19 (19.6)		6 (20.0)	2 (10.0)	8 (16.0)	
20–39	26 (43.3)	17 (45.9)	43 (44.3)		13 (43.3)	9 (45.0)	22 (44.0)	
40–59	10 (16.7)	12 (32.4)	22 (22.7)		6 (20.0)	7 (35.0)	13 (26.0)	
60 years or older	6 (10.0)	6 (16.2)	12 (12.4)		5 (16.7)	2 (10.0)	7 (14.0)	
Education level				0.016				0.429
Illiterate	17 (28.3)	3 (8.1)	20 (20.6)		10 (33.3)	3 (15.0)	13 (26.0)	
Incomplete ES	30 (50.0)	15 (40.5)	45 (46.4)		16 (53.3)	11 (55.0)	27 (54.0)	
Complete ES	5 (8.3)	5 (13.5)	10 (10.3)		1 (3.3)	1 (5.0)	2 (4.0)	
Incomplete HS	0 (0.0)	0 (0.0)	0 (0.0)		0 (0.0)	1 (5.0)	1 (2.0)	
Complete HS	0 (0.0)	1 (2.7)	1 (1.0)		0 (0.0)	1 (5.0)	1 (2.0)	
Complete Higher Education	1 (1.7)	0 (0.0)	1 (1.0)		0 (0.0)	0 (0.0)	0 (0.0)	
No Information	7 (11.7)	13 (35.1)	20 (20.6)		3 (10.0)	3 (15.0)	6 (12.0)	
Ethnicity				0.281				0.375
Kaiowá	32 (53.3)	18 (48.6)	50 (51.5)		17 (56.7)	10 (50.0)	27 (54.0)	
Guarani	14 (23.3)	5 (13.5)	19 (19.6)		6 (20.0)	2 (10.0)	8 (16.0)	
Terena	12 (20.0)	10 (27.0)	22 (22.7)		7 (23.3)	8 (40.0)	15 (30.0)	
Kadiwéu	1 (1.7)	0 (0.0)	1 (1.0)		0 (0.0)	0 (0.0)	0 (0.0)	
Ofaié	0 (0.0)	1 (2.7)	1 (1.0)		0 (0.0)	0 (0.0)	0 (0.0)	
Not indigenous	1 (1.7)	3 (8.1)	4* (4.1)		0 (0.0)	0 (0.0)	0 (0.0)	

F: female; M: male; ES: elementary school; HS: high school

The highest percentages of HIV and AIDS infection were found in indigenous people with incomplete elementary education, represented by 45 (46.4%) and 27 (54.0%), respectively, followed by illiterates, represented by 20 (20.6%) and 13 cases (26.0%), respectively, with statistical significance for HIV cases (p = 0.016). Still regarding schooling, we identified a high percentage of records without such information – 20.6% for HIV and 12.0% for AIDS ( Table 2 ).

Of the eight ethnic groups served by the DSEI-MS, five had registered cases: Kaiowá, Terena, Guarani, Kadwéu and Ofaié, as well as non-indigenous individuals living in the villages. The Kaiowá ethnic group had the highest concentration of HIV infection, with 50 cases (51.5%), followed by the Terena ethnic group with 22 cases (22.7%). The same ethnic groups were also predominant for AIDS cases, with 27 (54.0%) and 15 cases (30.0%), respectively ( Table 2 ).

Although the HIV and AIDS detection rates were higher for the Guarani and Terena ethnic groups, we must highlight the Ofaié ethnic group, whose rates were considerably higher than the other when considering their small population size. Mortality and fatality rates were also higher for the Kaiowá when compared to the other ethnic groups. Moreover, deaths were recorded only for the Guarani and Terena ethnic groups. Although the Terena had the lowest mortality rate, they had the second highest fatality rate ( Table 3 ).

**Table 3:** Accumulated HIV and AIDS detection rates, number of AIDS deaths, mortality rate and fatality rate, according to ethnic group. Indigenous Special Health District Mato Grosso do Sul. Mato Grosso do Sul, Brazil, 2001–2014.

Ethnicity	Base population in 2014	HIV detection rate (per 100 thousand inhabitants)	AIDS detection rate (per 100 thousand inhabitants)	Deaths by AIDS (n)	Mortality rate (per 100 thousand inhabitants)	Fatality rate (%)
Kaiowá	34,030	146.9	79.3	22	64.6	40.7
Terena	23,491	93.7	63.9	6	25.5	25
Guarani	11,371	167.1	70.4	3	26.4	15.8
Kadiwéu	1,365	73.3	0.0	0	0	0
Ofaié	54	1851.9	0.0	0	0	0
Not indigenous	602	664.5	0.0	0	0	0

Total	73,181	133.9	68.3	31	42.4	30.1

Comparisons of detection, mortality and fatality rates between the two analyzed periods (2001 to 2007 and 2008 to 2014) showed that the AIDS detection rate increased more than that of HIV infection. Furthermore, the mortality rate by AIDS increased, whereas the fatality decreased. Of the Health Base Poles with highest detection rates of HIV and AIDS cases, Iguatemi was the only one that showed a decrease in both (HIV and AIDS). In contrast, the Amambai Center presented increased detection rates for both HIV and AIDS. The AIDS detection rate increased in the Dourados Center (25.4/100 thousand for 72.4/100 thousand) whereas its mortality and fatality decreased ( Table 4 ).

**Table 4:** Accumulated rates of HIV and AIDS detection, and of AIDS mortality and fatality in the three Health Base Poles with the highest number of cases. Indigenous Special Health District Mato Grosso do Sul. Mato Grosso do Sul, Brazil, numbers of 2007 and 2014.

Health Base Poles	Base population	HIV (n)	HIV det rate (per 100,000 inhabitants)	AIDS (n)	AIDS det rate (per 100,000 inhabitants)	Deaths by AIDS (n)	Mortality rate (per 100,000 inhabitants)	Fatality rate (%)
2001–2007								

Dourados	11,825	16	135.3	3	25.4	8	67.7	47.1
Amambai	11,105	0	0.0	1	9.0	1	9.0	100.0
Iguatemi	4,186	7	167.2	3	71.7	1	23.9	14.3
DSEI-MS	63,594	34	53.5	10	15.7	13	20.4	35.1

2008–2014								

Dourados	15,186	17	111.9	11	72.4	4	26.3	21.1
Amambai	12,953	22	169.8	14	108.1	9	69.5	40.9
Iguatemi	5,106	5	97.9	2	39.2	1	19.6	20.0
DSEI-MS	73,181	63	86.1	40	54.7	18	24.6	27.3

Det rate: detection rate; DSEI-MS: Indigenous Special Health District Mato Grosso do Sul.

## DISCUSSION

This study showed the evolution of the detection rates of HIV infection and AIDS in the indigenous population of Mato Grosso do Sul. Women in the 20 to 39 years age group, with lower schooling and of the Kaiowá ethnic group presented the highest HIV and AIDS infection percentages. The Kaiowá, Terena and Guarani ethnic groups were the most exposed one, with records of high HIV infection and AIDS rates. These results also contributed to the high mortality and fatality rates in these groups. The Health Base Poles of Amambai, Dourados and Iguatemi, located in the southern region of the state, concentrated around 70% of cases of HIV infection and AIDS.

Oscillations in detection rates in the study period may be more strongly associated with the diagnostic ability of services than to the epidemiological behavior of the virus transmission or disease burden on the population. These must be interpreted as a warning sign for the health care services for the state’s indigenous peoples.

The Guarani and Kaiowá have a very complex health picture, with high prevalence of infectious diseases, high infant mortality and malnutrition, as well as the increasing prevalence of chronic non-communicable diseases^7^ . The historical context of the contact of these peoples with the non-indigenous society, their geographic location along a border, the proximity and ease of access between villages and urban centers – something common in Mato Grosso do Sul’s villages –, associated with social problems such as unemployment, violence, exploitation and sexual abuse^14^ indicate a situation of great vulnerability that may help to understand the concentration of two-thirds of the cases identified in this study between these two ethnic groups, especially in the villages belonging to the Dourados, Amambai and Iguatemi Health Base Poles.

Furthermore, the mobility between villages – even outside the Brazilian territory since the Guarani and Kaiowá territories extend to Paraguay^8 , 9^ – hinders the regular access to health services and diagnosis. Situations of intense inter-ethnic contact and marginalization in the access to health services are also risk factors for the circulation and spread of HIV. Socioeconomic, environmental, and cultural determinants interfere with the intensity of disease spread, making prevention and control activities complex, as evidenced in a study on the introduction of AIDS in the Xokleng people of Santa Catarina^17^_._

In this study, the population with the greatest vulnerability to the spread of HIV and AIDS were young people, especially women with low schooling level. Unfavorable socioeconomic conditions are associated with greater exposure to risk factors. Schooling is a social status marker and has effects on the access to information and knowledge about disease prevention methods and its treatment^18 , 19^ , including for indigenous peoples. Studies have shown that schooling is also directly associated with the survival of patients with HIV and AIDS, having a protective effect against most causes of death^20 , 21^ .

The findings of this study differ from those observed among the indigenous peoples of Peru and Bolivia, whose HIV infection/AIDS ratio among men and women was 3:1 and 5:3, respectively^19^ . Another study on sexually transmitted infections, also conducted on the indigenous population of Mato Grosso do Sul between 2001 and 2005, first identified the prevalence of AIDS among men up to 2002, and in the following years, the number of cases between sexes was matched^22^ . However, these results do not allow us to affirm that a “feminization” of HIV and AIDS cases has occurred among indigenous peoples in Mato Grosso do Sul because, as has been observed in Brazil^23^ , such rates may be related to the greater coverage of women’s health provided by the Programa Estadual de Proteção à Gestante (State Protection Program for Pregnant Women), which conducts HIV screening tests during pregnancy.

On the other hand, the limitations of the diagnosis of HIV infection in the male indigenous population may be associated with the low coverage of diagnostic tests, absence of men in the villages for work-related reasons, and reduced male demand for health services. The low diagnostic coverage favors the spread of the infection since ignorance about the disease leads to the continuation of sexual practices without prevention. The gradual inclusion of rapid HIV testing in villages as of 2012 may have contributed to the resumption in the growth of the HIV detection rate in the last two years of the study period since it led to increased diagnostic coverage, as observed in other DSEIs across Brazil^24^ . The predominance of AIDS cases diagnosed in the 20 to 39 years and 40 to 60 years age groups was also found in the Brazilian scenario^b^ and in a study conducted among the indigenous peoples of Bolivia and Peru, which found one in two cases among 20 and 39 years^19^ .

The observation of mortality and fatality rates among the Kaiowá is relevant for the organization of health services since it indicates that this ethnic group has less access to antiretroviral therapy. The decrease in mortality and fatality rates in the Dourados Base in the second period suggests a greater structuring of health services in the villages, and articulation with specialized care services (SAE) for the treatment of indigenous individuals with AIDS. The differentiated behavior of the HIV and AIDS rates in each of the Health Base Pole shows the need for the separate evaluation of health services, given that these present different structuring forms to address the problem.

The main limitations of this study are related to the use of secondary data sources, which compromise the quality of information. The deficiency in the completeness of the variable indigenous race/skin color in Sinan and the underreporting of HIV and AIDS cases in indigenous peoples are factors that influence the number of identified cases and, consequently, information coverage.

This study used daily EMSI records as a data source to improve the coverage of HIV infection cases, since compulsory notification was only included in Sinan in 2005 for pregnant women^g^ , and in 2014 for the general population^h^ . Underreporting and incompleteness of the variable race/skin color data make it difficult to characterize health inequity situations, and to establish strategies for coping with such inequities^25 , 26^ .

Despite these limitations, this study has brought benefits by addressing a disease that is not very well known in the epidemiology of indigenous peoples in Brazil, which impacts on the morbidity and mortality profile, and is related to the emergence of sexually transmitted infections and its association with social and cultural changes resulting from the process of interaction with the non-indigenous society.

The challenge of working with sexually transmitted diseases in an inter-ethnic context implies the need to comprehend sexuality values inherent in each indigenous group^27^ and the articulation between indigenous and non-indigenous health systems^28^ . Knowledge of logistical difficulties, population distribution and more vulnerable areas help to define strategies to expand the diagnostic coverage and facilitate the access to treatment^29^ . In this sense, the training of human resources to work in intercultural contexts is fundamental to increase response capacity Sasi-SUS in disease prevention and treatment actions.

This study showed that the endemic character of HIV and AIDS – with prevalence of HIV infection in the southern region of the state –, may become epidemic in some years since there are cases in other indigenous areas of Mato Grosso do Sul. Among the crucial intervention measures to address the problem, we can cite the expansion of diagnostic coverage, the guarantee of access to treatment, and measures to prevent HIV transmission.

## Footnotes

a UNAIDS. Prevention Gap Report. Geneva; 2016 [cited 1 Jun 2017]. Available from http://www.unaids.org/sites/default/files/media_asset/2016-prevention-gap-report_en.pdf

b Ministério da Saúde (BR), Secretaria de Vigilância em Saúde, Departamento de IST, Aids e Hepatites Virais. Boletim epidemiológico HIV/Aids. Brasília, DF; 2017 [cited 7 May 2018]. Available from: http://www.aids.gov.br/pt-br/pub/2017/boletim-epidemiologico-hivaids-2017

d Brasil. Lei 9.836, de 23 de setembro de 1999. Acrescenta dispositivos à Lei n8.080, de 19 de setembro de 1990, que “dispõe sobre as condições para a promoção, proteção e recuperação da saúde, a organização e o funcionamento dos serviços correspondentes e dá outras providências”, instituindo o Subsistema de Atenção à Saúde Indígena. Brasília, DF; 1999 [cited 10 Sep 2018]. Available from: http://www.planalto.gov.br/ccivil_03/leis/L9836.htm

e Instituto Brasileiro de Geografia e Estatística. Os indígenas no censo demográfico 2010. Brasília, DF: IBGE; 2010. [cited 2017 Apr 16]. Available from: http://www.ibge.gov.br/indigenas/indigena_censo2010.pdf

f Ministério da Saúde (BR), Secretaria Especial de Saúde Indígena, Distrito Sanitário Especial Indígena Mato Grosso do Sul. Caracterização demográfica, étnico-cultural dos povos indígenas do DSEI Mato Grosso do Sul. Campo Grande, MS: DSEI/MS; 2014.

g Ministério da Saúde (BR), Secretaria de Vigilância em Saúde. Portaria nº 33, de 14 de julho de 2005. Inclui doenças à relação de notificação compulsória, define agravos de notificação imediata e a relação dos resultados laboratoriais que devem ser notificados pelos Laboratórios de Referência Nacional ou Regional. Brasília, DF; 2005 [cited 24 Aug 2018]. Available from: http://bvsms.saude.gov.br/bvs/saudelegis/svs/2005/prt0033_14_07_2005.html

h Ministério da Saúde (BR). Portaria nº 1.271, de 6 de junho de 2014. Define a Lista Nacional de Notificação Compulsória de doenças, agravos e eventos de saúde pública nos serviços de saúde públicos e privados em todo o território nacional, nos termos do anexo, e dá outras providências. Brasília, DF; 2014 [cited 24 Aug 2018]. Available from: http://bvsms.saude.gov.br/bvs/saudelegis/gm/2014/prt1271_06_06_2014.html
