# Supplementary material for: Epidemiological aspects of HIV infection and AIDS among indigenous populations
Source: Rev Saude Publica. 2019 Sep 2;53:71. doi: 10.11606/S1518-8787.2019053000362 (PMC6758848; doi:10.11606/S1518-8787.2019053000362)
Supplement: Translation

# Translation: Aspectos epidemiológicos da infecção pelo HIV e da aids entre povos indígenas

**Authors:** Samara Vilas-Bôas Graeff, Renata Palópoli Pícolli, Rui Arantes, Vivianne de Oliveira Landgraf de Castro, Rivaldo Venâncio da Cunha

**Affiliations:** I Prefeitura Municipal de Campo Grande. Secretaria Municipal de Saúde. Campo Grande, MS, Brasil; II Fundação Oswaldo Cruz de Mato Grosso do Sul. Campo Grande, MS, Brasil; III Fundação Oswaldo Cruz. Escola Nacional de Saúde Pública Sérgio Arouca. Departamento de Endemias. Rio de Janeiro, RJ, Brasil; IV Universidade Federal de Mato Grosso do Sul. Laboratório de Imunologia Clínica. Faculdade de Ciências Farmacêuticas, Alimentos e Nutrição. Campo Grande, MS, Brasil; V Universidade Federal de Mato Grosso do Sul. Faculdade de Medicina. Campo Grande, MS, Brasil

**Keywords:** Saúde de Populações Indígenas, Infecções por HIV, epidemiologia, Síndrome de Imunodeficiência Adquirida, epidemiologia, Soroprevalência de HIV, tendências, Sorodiagnóstico da AIDS, tendências

## Abstract

**OBJETIVO:**

Descrever os aspectos epidemiológicos da infecção pelo HIV e da aids entre povos indígenas do Mato Grosso do Sul.

**MÉTODOS:**

Estudo epidemiológico descritivo sobre ocorrência e distribuição da infecção pelo HIV e aids na população indígena assistida pelo Distrito Sanitário Especial Indígena Mato Grosso do Sul, entre 2001 e 2014, a partir de três bases de dados secundários. Calcularam-se as taxas anuais de detecção e de prevalência de HIV e aids, com distribuição dos casos segundo aldeia, Polo Base e variáveis sociodemográficas. As taxas acumuladas de detecção, mortalidade e letalidade foram calculadas por etnia e para os Polos Base com o maior número de casos.

**RESULTADOS:**

A taxa de detecção de HIV flutuou entre 0,0 e 18,0/100 mil pessoas no período. Para a aids, não houve notificação antes de 2007, mas em 2012 sua taxa chegou a 16,6/100 mil. A prevalência de HIV indicou crescimento entre 2001 e 2011, e para a aids observou-se aumento contínuo a partir de 2007. As maiores taxas de detecção de HIV ocorreram entre os Guarani (167,1/100 mil) e de aids, entre os Kaiowá (79,3/100 mil); as taxas de mortalidade e letalidade foram superiores entre os Kaiowá. Para o Polo Base de Dourados, observou-se elevação da taxa de detecção de aids e diminuição das taxas de mortalidade e letalidade.

**CONCLUSÕES:**

A infecção pelo HIV e a aids mostraram-se crescentes entre povos indígenas, com distribuição da doença principalmente nos Polos Base da região sul do estado, onde observa-se também maior vulnerabilidade econômica e social. O caráter endêmico do HIV e da aids pode se tornar epidêmico em alguns anos, considerando a existência de casos em outras aldeias do estado. Sua ocorrência entre os Guarani e Kaiowá sinaliza a necessidade de ampliação do diagnóstico, do acesso ao tratamento e de medidas de prevenção.

## INTRODUÇÃO

A síndrome da imunodeficiência adquirida (aids) já acometeu milhões de pessoas em todo o mundo. Em 2015, cerca de 36,7 milhões viviam com o vírus^a^ . No Brasil, de 1980 até junho de 2017 foram contabilizados 882.810 casos de aids^b^ . Entre 2006 e 2016, observou-se no país uma tendência de estabilização da taxa de detecção de aids, com uma média de 18,5/100 mil habitantes em 2016. Contudo, no estado de Mato Grosso do Sul, houve crescimento de 8,2% no período, representado por uma taxa de 19,8/100 mil habitantes em 2016, a maior da região Centro-Oeste^b^ .

Entre os povos indígenas, o impacto da aids ainda é pouco conhecido. Estudos, entretanto, têm destacado a maior vulnerabilidade desses grupos populacionais à transmissão do HIV, relacionada às piores condições de vida, menor nível socioeconômico, educacional, exclusão social e dificuldade de acesso aos serviços de saúde^1 , 2^ . As informações oriundas de países como Estados Unidos da América, Canadá, Nova Zelândia e Austrália indicam um aumento expressivo da taxa de detecção de HIV entre os povos indígenas nas últimas décadas^3^ . Em geral, essa taxa é maior entre indígenas do que entre não indígenas, o que indica as iniquidades em saúde, no que diz respeito ao acesso ao diagnóstico e tratamento da doença^1 , 2 , 5^ .

No Brasil, o primeiro caso de aids em indígenas foi registrado em 1987, e até 1999 conheciam-se somente 33 casos. Já entre 2000 e junho de 2017, o número aumentou para 1.042^b^ . Contudo, o impacto da aids em povos indígenas não pode ser caracterizado apenas por números absolutos e/ou taxas. As dimensões socioculturais, econômicas, demográficas, geográficas de cada povo indígena, associadas ao processo de interação com a sociedade não indígena, os expõem a fatores de risco singulares que levam à maior vulnerabilidade em relação aos demais grupos populacionais brasileiros^1 , 6 , c^ .

A oferta de serviços de saúde para os indígenas também repercute neste cenário. No Brasil, em seu histórico recente, a atenção à saúde dos povos indígenas ganhou força em 1999 com a criação do Subsistema de Atenção à Saúde Indígena, no âmbito do Sistema Único de Saúde (Sasi-SUS)^d^ .

Considerando a necessidade de investigações que supram as lacunas relacionadas à ocorrência de HIV e aids entre indígenas e que possam subsidiar a formulação de políticas de assistência qualificadas direcionadas a essa população, este estudo teve por objetivo descrever os aspectos epidemiológicos desses casos entre os povos indígenas do estado de Mato Grosso do Sul, no período de 2001 a 2014.

## MÉTODOS

Estudo descritivo sobre a ocorrência e distribuição dos casos de infecção pelo HIV e aids na população indígena do estado de Mato Grosso do Sul assistida pelo Distrito Sanitário Especial Indígena Mato Grosso do Sul (DSEI-MS), no período de 2001 a 2014. Mato Grosso do Sul tem a segunda maior população indígena do Brasil, com 73.295 pessoas autodeclaradas^e^ . O DSEI-MS possui 14 Polos Base, primeira referência para as equipes multidisciplinares de saúde indígena (EMSI) que atuam nas aldeias e atendem 70.032 pessoas de oito etnias (Kaiowá, Guarani Ñandeva, Terena, Kadiwéu, Kinikinau, Guató, Ofaié e Atikun) distribuídas em 75 aldeias e 26 acampamentos, localizados em 31 dos 79 municípios do estado^f^ .

Neste estudo, foram incluídos indivíduos com diagnósticos de infecção pelo HIV, oriundos dos registros dos atendimentos diários das EMSI, e de aids, provenientes do Sistema de Informação de Agravos de Notificação (Sinan), selecionados pela variável raça/cor indígena, que residiam em aldeias e acampamentos indígenas do estado. Excluíram-se os casos duplicados, os indígenas residentes em aldeias/áreas urbanas não assistidas pelo DSEI-MS e as crianças expostas ao HIV na gestação.

Os dados registrados pelas EMSI no decorrer do período do estudo foram obtidos de junho de 2014 a janeiro de 2015 por meio da vinculação manual do nome, data de nascimento e nome da mãe entre três bases de dados: 1. registros provenientes dos atendimentos diários das EMSI; 2. banco de dados do Sinan; 3. Sistema de Informação de Atenção à Saúde Indígena (Siasi). Considerou-se local de residência aquele registrado no momento do diagnóstico de HIV pela EMSI ou o lançado no Sinan, para os casos de aids. As variáveis sociodemográficas (etnia, sexo, data de nascimento e escolaridade) foram obtidas no Siasi, assim como as bases populacionais utilizadas para os cálculos das taxas descritas em seguida.

Para calcular as taxas anuais de detecção de HIV e aids, utilizou-se o número de casos novos registrados de cada um. As pessoas diagnosticadas diretamente com aids foram consideradas somente para a taxa de detecção de aids. Para inclusão das pessoas diagnosticadas em áreas não assistidas pelo DSEI-MS, considerou-se o território para o qual a pessoa se mudou e o ano em que isso ocorreu.

Para o cálculo da prevalência de HIV, utilizou-se o número de casos antigos e novos de HIV de cada ano e excluíram-se os óbitos, os que evoluíram para aids e as pessoas que se mudaram das áreas atendidas pelo DSEI-MS. Para o cálculo da prevalência da aids, utilizou-se o número de casos antigos e os novos diagnósticos, mais os que evoluíram para aids naquele ano, excluídos os óbitos e pessoas que se mudaram das áreas em que o DSEI-MS prestava assistência.

A análise da associação entre os anos e a taxa de detecção de HIV e de aids, assim como as prevalências, foi realizada pelo teste do qui-quadrado de Pearson, com correção de Bonferroni, quando necessário, utilizando o programa estatístico IBM SPSS^®^, versão 24.0, com um nível de significância de 5%.

Os demais resultados foram apresentados na forma de estatística descritiva. A distribuição espacial dos casos de HIV e aids na área de abrangência do DSEI-MS foi determinada segundo local de residência (aldeia e Polo Base) no momento do diagnóstico. Calcularam-se também as taxas de detecção de HIV e de aids, assim como de mortalidade e letalidade por aids acumuladas referentes a todo o período, considerando a população total de cada etnia no ano de 2014. As taxas acumuladas também foram calculadas para os três Polos Base que concentraram a maioria dos diagnósticos, divididas em dois períodos (2001 a 2007, com população-base de 2007) e (2008 a 2014, com população-base de 2014).

O estudo recebeu aprovação da Comissão Nacional de Ética em Pesquisa (Conep) sob o Parecer 707.439/2014.

## RESULTADOS

As taxas de detecção de infecção pelo HIV apresentaram flutuações anuais no período do estudo, com valores que foram de zero até 18,0/100 mil pessoas. As maiores taxas foram registradas em 2006 (17,8/100 mil) e 2011 (18,0/100 mil). Em relação aos casos de aids, não houve registros da doença entre 2001 e 2006. A partir de 2007, observou-se um aumento até 2012, quando a taxa de detecção chegou a 16,6/100 mil. Em 2012 e 2013, as taxas de detecção de aids foram superiores às de HIV ( Figura ).

**Figura:** Taxas anuais (por 100 mil habitantes) de detecção (A), de prevalência (B) e respectivos intervalos de confiança (IC95%) de casos de infecção pelo HIV e aids na população indígena atendida pelo Distrito Sanitário Especial Indígena Mato Grosso do Sul. Mato Grosso do Sul, Brasil, 2001–2014.

As taxas anuais de prevalência de HIV apresentaram crescimento no período de 2001 a 2011 e uma diminuição a partir de 2011, caindo de 51,2/100 mil para 39,6/100 mil em 2014. Para a aids, a taxa de prevalência apresentou aumento contínuo a partir de 2007, aproximando-se gradativamente da prevalência de infecção pelo HIV depois de 2012 ( Figura ).

No período do estudo, registraram-se 97 casos de infecção pelo HIV e 50 de aids, envolvendo 103 pessoas, das quais 6 foram diagnosticadas diretamente com aids. Apesar de o HIV estar presente em 80% dos Polos Base e a aids em 64% deles, a distribuição dos casos foi bastante desigual. Os Polos de Dourados, Amambai e Iguatemi concentraram juntos 69% dos casos de infecção por HIV e 68% de aids. O Polo Base de Dourados sozinho registrou 34% dos casos de infecção pelo HIV. As aldeias Jaguapirú, Amambai, Taquapery e Porto Lindo registraram 52% dos casos de aids, 20% dos quais estavam na aldeia Jaguapiru (Polo Base de Dourados) ( Tabela 1 ).

**Tabela 1:** Distribuição dos casos de infecção pelo HIV e aids no momento do diagnóstico, por Polo Base e aldeia, e taxa de detecção acumulada (por 100 mil habitantes), por Polo Base, com população-base do ano de 2014. Distrito Sanitário Especial Indígena Mato Grosso do Sul. Mato Grosso do Sul, Brasil, 2001–2014.

Polo base	Aldeia (população de 2014)	HIV			Aids		
	
		Aldeia	Polo base	Tx det PB	Aldeia	Polo base	Tx det PB
					
		n (%)	n (%)	/100 mil	n (%)	n (%)	/100mil
Dourados	Bororó (6.341)	16 (16,5)	33 (34,0)	217,3	4 (8,0)	14 (28,0)	92,2
	Jaguapirú (6.753)	17 (17,5)			10 (20,0)		
Amambai	Amambai (7.031)	12 (12,4)	23 (23,7)	177,6	6 (12,0)	15 (30,0)	115,8
	Limão Verde (1.602)	4* (4,1)			4 (8,0)		
	Taquapery (3.089)	7 (7,2)			5 (10,0)		
Iguatemi	Porto Lindo (3.946)	11* (11,3)	11 (11,3)	215,4	5 (10,0)	5 (10,5)	97,9
Aquidauana	Aldeinha (398)	1 (1,0)	7 (7,2)	103,4	0 (0,0)	3 (6,0)	44,3
	Bananal (1.109)	3 (3,1)			2 (4,0)		
	Limão Verde (1.161)	2* (2,1)			0 (0,0)		
	Ypegue (736)	1 (1,0)			1 (2,0)		
Caarapó	Caarapó (4.694)	5 (5,2)	6 (6,2)	95,4	4 (8,0)	4 (8,0)	63,6
	Taquara (283)	1 (1,0)			0 (0,0)		
Miranda	Moreira (1.123)	1 (1,0)	5 (5,2)	68,0	1 (2,0)	3 (6,0)	40,8
	Passarinho (1.221)	4 (4,1)			2 (4,0)		
Paranhos	Pirajuí (2.219)	3 (3,1)	3 (3,1)	60,9	1 (2,0)	1 (2,0)	20,3
Sidrolândia	Buriti (953)	2 (2,1)	3 (3,1)	67,6	1 (2,0)	2 (4,0)	45,1
	Córrego do Meio (574)	1 (1,0)			1 (2,0)		
Tacuru	Sassoró (2.111)	3*(3,1)	3 (3,1)	99,2	3* (6,0)	3 (6,0)	99,2
Brasilândia	Ofaié (105)	2 (2,1)	2 (2,1)	1904,8	0 (0,0)	0 (0,0)	0,0
Bodoquena	Alves de Barros (839)	1 (1,0)	1 (1,0)	103,3	0 (0,0)	0 (0,0)	0,0

Total		97 (100,0)	97 (100,0)	132,5	50 (100,0)	50 (100,0)	68,3

Tx det PB: taxa de detecção por Polo Base

* Inserido um (1) caso diagnosticado em pessoa que residia em um município no momento do diagnóstico e que posteriormente se mudou para a aldeia no período do estudo.

Em relação às taxas de detecção de HIV e aids acumuladas por Polo Base, observaram-se algumas diferenças ordinais na distribuição. O Polo Base de Brasilândia obteve a maior taxa de detecção de HIV (1.904,8/100 mil), mas é necessário considerar seu tamanho populacional. Em seguida vieram os Polos de Dourados (217,3/100 mil), Iguatemi (215,4/100 mil) e Amambai (177,6/100 mil). Para os casos de aids, a taxa de detecção foi maior para os Polos de Amambai (115,8/100 mil), Tacuru (99,2/100 mil) e Iguatemi (97,9/100 mil), deixando o Polo de Dourados fora dos três primeiros colocados ( Tabela 1 ).

A faixa etária de maior ocorrência de infecção pelo HIV e aids foi de 20 a 39 anos, com 43 (44,3%) e 22 (44,0%) casos, respectivamente. Houve significância estatística para os casos de HIV, cujo valor de p foi 0,040. Na distribuição por sexo, verificou-se que a maior parte dos casos de HIV e aids ocorreu entre as mulheres, correspondendo a 60 (61,9%) e 30 (60%) casos, nessa ordem. Nota-se que a infecção pelo HIV ocorreu mais precocemente no sexo feminino do que no masculino, visto que, na faixa etária de 10 a 19 anos, a razão entre os sexos foi de aproximadamente nove mulheres para cada homem ( Tabela 2 ).

**Tabela 2:** Distribuição dos casos de infecção pelo HIV e aids, segundo variáveis sociodemográficas. Distrito Sanitário Especial Indígena Mato Grosso do Sul. Mato Grosso do Sul, Brasil, 2001–2014.

Variável	HIV				Aids			
	
	Sexo			p	Sexo			p
	
	F	M	Total		F	M	Total	
					
	n (%)	n (%)	n (%)		n (%)	n (%)	n (%)	
Faixa etária (anos)				0,040				0,536
0–9	1 (1,7)	0 (0,0)	1 (1,0)		0 (0,0)	0 (0,0)	0 (0,0)	
10–19	17 (28,3)	2 (5,4)	19 (19,6)		6 (20,0)	2 (10,0)	8 (16,0)	
20–39	26 (43,3)	17 (45,9)	43 (44,3)		13 (43,3)	9 (45,0)	22 (44,0)	
40–59	10 (16,7)	12 (32,4)	22 (22,7)		6 (20,0)	7 (35,0)	13 (26,0)	
60 ou mais	6 (10,0)	6 (16,2)	12 (12,4)		5 (16,7)	2 (10,0)	7 (14,0)	
Escolaridade				0,016				0,429
Analfabeto	17 (28,3)	3 (8,1)	20 (20,6)		10 (33,3)	3 (15,0)	13 (26,0)	
E. F. incompleto	30 (50,0)	15 (40,5)	45 (46,4)		16 (53,3)	11 (55,0)	27 (54,0)	
E. F. completo	5 (8,3)	5 (13,5)	10 (10,3)		1 (3,3)	1 (5,0)	2 (4,0)	
E. M. incompleto	0 (0,0)	0 (0,0)	0 (0,0)		0 (0,0)	1 (5,0)	1 (2,0)	
E. M. completo	0 (0,0)	1 (2,7)	1 (1,0)		0 (0,0)	1 (5,0)	1 (2,0)	
Superior completo	1 (1,7)	0 (0,0)	1 (1,0)		0 (0,0)	0 (0,0)	0 (0,0)	
Sem informação	7 (11,7)	13 (35,1)	20 (20,6)		3 (10,0)	3 (15,0)	6 (12,0)	
Etnia				0,281				0,375
Kaiowá	32 (53,3)	18 (48,6)	50 (51,5)		17 (56,7)	10 (50,0)	27 (54,0)	
Guarani	14 (23,3)	5 (13,5)	19 (19,6)		6 (20,0)	2 (10,0)	8 (16,0)	
Terena	12 (20,0)	10 (27,0)	22 (22,7)		7 (23,3)	8 (40,0)	15 (30,0)	
Kadiwéu	1 (1,7)	0 (0,0)	1 (1,0)		0 (0,0)	0 (0,0)	0 (0,0)	
Ofaié	0 (0,0)	1 (2,7)	1 (1,0)		0 (0,0)	0 (0,0)	0 (0,0)	
Não indígena	1 (1,7)	3 (8,1)	4 (4,1)		0 (0,0)	0 (0,0)	0 (0,0)	

F: feminino; M: masculino; E. F.: ensino fundamental; E. M.: ensino médio

Os maiores percentuais de infecção pelo HIV e aids foram encontrados em indígenas com ensino fundamental incompleto, representados por 45 (46,4%) e 27 casos (54,0%), respectivamente, seguidos pelos analfabetos, representados por 20 (20,6%) e 13 casos (26,0%), respectivamente, com significância estatística (p = 0,016) para os casos de HIV. Ainda sobre a escolaridade, identificou-se um elevado percentual de registros sem informação – 20,6% para HIV e 12,0% para aids ( Tabela 2 ).

Das oito etnias atendidas pelo DSEI-MS, cinco tiveram casos registrados: Kaiowá, Terena, Guarani, Kadwéu e Ofaié, além de não indígenas residentes em aldeias. A etnia Kaiowá apresentou maior concentração de infecção pelo HIV, com 50 casos (51,5%), seguida da etnia Terena, com 22 casos (22,7%). Para os casos de aids, também observou-se predomínio das mesmas etnias, com 27 (54,0%) e 15 casos (30,0%), respectivamente ( Tabela 2 ).

Ainda que as taxas de detecção de HIV e aids tenham sido maiores para as etnias Guarani e Terena, merece destaque a etnia Ofaié, cujas taxas foram consideravelmente superiores às anteriores, devido a sua pequena representatividade populacional. As taxas de mortalidade e letalidade foram igualmente superiores para os Kaiowá quando comparadas às de outras etnias. Além dessa, óbitos foram registrados apenas para as etnias Guarani e Terena. Apesar de os Terena terem apresentado a menor taxa de mortalidade, tiveram a segunda maior letalidade ( Tabela 3 ).

**Tabela 3:** Taxas acumuladas de detecção de HIV e aids, número de óbitos por aids, taxa de mortalidade e taxa de letalidade, segundo etnia. Distrito Sanitário Especial Indígena Mato Grosso do Sul. Mato Grosso do Sul, Brasil, 2001–2014.

Etnia	População-base em 2014	Taxa de detecção de HIV (por 100 mil habitantes)	Taxa de detecção de aids (por 100 mil habitantes)	Óbitos por aids (n)	Taxa de mortalidade (por 100 mil habitantes)	Taxa de letalidade (%)
Kaiowá	34.030	146,9	79,3	22	64,6	40,7
Terena	23.491	93,7	63,9	6	25,5	25
Guarani	11.371	167,1	70,4	3	26,4	15,8
Kadiwéu	1.365	73,3	0,0	0	0	0
Ofaié	54	1851,9	0,0	0	0	0
Não indígena	602	664,5	0,0	0	0	0

Total	73.181	133,9	68,3	31	42,4	30,1

As comparações das taxas de detecção, de mortalidade e letalidade entre os dois períodos analisados (2001 a 2007 e 2008 a 2014) indicaram que a taxa de detecção de aids aumentou mais que a de infecção pelo HIV. Além disso, houve um aumento na taxa de mortalidade por aids e uma diminuição na letalidade. Dos Polos Base com maior taxa de detecção de casos de HIV e aids, Iguatemi foi o único que apresentou diminuição de ambas as taxas de detecção (HIV e aids). Em contrapartida, o Polo de Amambai mostrou aumento das taxas de detecção, tanto de HIV como de aids. Para o Polo de Dourados, observou-se elevação da taxa de detecção de aids (25,4/100 mil para 72,4/100 mil) e, por outro lado, diminuição de suas taxas de mortalidade e letalidade ( Tabela 4 ).

**Tabela 4:** Taxas acumuladas de detecção de HIV e aids, de mortalidade e de letalidade por aids nos três Polos Base com o maior número de casos. Distrito Sanitário Especial Indígena Mato Grosso do Sul. Mato Grosso do Sul, Brasil, cortes de 2007 e 2014.

Polo base	População-base	HIV (n)	Tx det HIV (por 100 mil habitantes)	Aids (n)	Tx det aids (por 100 mil habitantes)	Óbitos por aids (n)	Tx de mortalidade (por 100 mil habitantes)	Tx de letalidade (%)
2001–2007								

Dourados	11.825	16	135,3	3	25,4	8	67,7	47,1
Amambai	11.105	0	0,0	1	9,0	1	9,0	100,0
Iguatemi	4.186	7	167,2	3	71,7	1	23,9	14,3
DSEI-MS	63.594	34	53,5	10	15,7	13	20,4	35,1

2008–2014								

Dourados	15.186	17	111,9	11	72,4	4	26,3	21,1
Amambai	12.953	22	169,8	14	108,1	9	69,5	40,9
Iguatemi	5.106	5	97,9	2	39,2	1	19,6	20,0
DSEI-MS	73.181	63	86,1	40	54,7	18	24,6	27,3

Tx: taxa; Tx det: taxa de detecção; DSEI-MS: Distrito Sanitário Especial Indígena Mato Grosso do Sul.

## DISCUSSÃO

O presente estudo demonstrou evolução das taxas de detecção de infecção pelo HIV e aids na população indígena de Mato Grosso do Sul. As mulheres na faixa etária de 20 a 39 anos, com menor escolaridade e da etnia Kaiowá apresentaram maiores percentuais de infecção pelo HIV e aids. As etnias Kaiowá, Terena e Guarani foram as mais expostas, com registros de altas taxas de infecção pelo HIV e aids. Esses resultados contribuíram, também, para as elevadas taxas de mortalidade e letalidade nesses grupos. Os Polos Base de Amambai, Dourados e Iguatemi, na região sul do estado, concentraram em torno de 70% dos casos de infecção pelo HIV e de aids.

As oscilações nas taxas de detecção no período podem estar mais fortemente associadas à capacidade dos serviços para realizar diagnósticos e menos ao comportamento epidemiológico da transmissão do vírus ou à carga da doença na população. Elas devem ser encaradas como um sinal de alerta para os serviços de atenção à saúde dos povos indígenas do estado.

Os Guarani e Kaiowá apresentam um quadro de saúde bastante complexo, com alta prevalência de doenças infecciosas, elevada mortalidade infantil e desnutrição, juntamente com a prevalência crescente de doenças crônicas não transmissíveis^7^ . O contexto histórico de contato desses povos com a sociedade não indígena, a sua localização geográfica em uma faixa de fronteira, a proximidade e facilidade de acesso entre as aldeias e os centros urbanos, muito comuns nas aldeias do Mato Grosso do Sul, associados aos problemas sociais como desemprego, violência, exploração e abuso sexual^14^ , indicam uma situação de grande vulnerabilidade que pode ajudar a entender a concentração de dois terços dos casos identificados neste estudo entre essas duas etnias, em especial nas aldeias pertencentes aos Polos Base de Dourados, Amambai e Iguatemi.

Além disso, a mobilidade entre aldeias, inclusive para fora do território nacional, uma vez que o território Guarani e Kaiowá se estende para o Paraguai^8 , 9^ , dificulta o acesso regular aos serviços de saúde e o diagnóstico. Situações de intenso contato interétnico e a marginalização no acesso aos serviços de saúde também constituem fatores de risco para a circulação e propagação do HIV. Os determinantes socioeconômicos, ambientais e culturais interferem na intensidade de propagação da doença e tornam complexas as atividades de prevenção e controle, como evidenciado em estudo sobre a introdução da aids no povo Xokleng de Santa Catarina^17^_._

No presente estudo, a população com maior situação de vulnerabilidade à propagação do HIV e aids foi a de jovens, principalmente as mulheres e com baixa escolaridade. Condições socioeconômicas desfavoráveis estão associadas à maior exposição aos fatores de risco. A escolaridade é um marcador de condição social e repercute no acesso à informação e no conhecimento de métodos de prevenção da doença e de seu tratamento^18 , 19^ , inclusive para os povos indígenas. Estudos demonstraram que a escolaridade também está diretamente associada à sobrevida de pacientes com HIV e aids, tendo um efeito protetor contra a maioria das causas de óbito^20 , 21^ .

Os achados do presente estudo diferem do observado entre os povos indígenas do Peru e Bolívia, cuja razão de infecção pelo HIV/aids entre homens e mulheres foi de 3:1 e 5:3, respectivamente^19^ . Já outro estudo sobre infecções sexualmente transmissíveis também realizado com a população indígena de Mato Grosso do Sul entre 2001 e 2005 identificou primeiramente o predomínio de aids entre os homens até 2002, e nos anos seguintes equiparação de casos entre os sexos^22^ . Entretanto, tais resultados não permitem afirmar que ocorreu uma “feminilização” dos casos de HIV e aids entre indígenas no Mato Grosso do Sul, como vem sendo observado no Brasil^23^ , pois podem estar relacionados à maior cobertura da assistência às mulheres proporcionada pelo Programa Estadual de Proteção à Gestante, que realiza exames de detecção de HIV na gestação.

Por outro lado, as limitações do diagnóstico da infecção pelo HIV na população indígena masculina podem estar associadas à baixa cobertura de exames diagnósticos, ausência dos homens nas aldeias por motivos laborais e reduzida procura masculina pelos serviços de saúde. A baixa cobertura diagnóstica favorece a disseminação da infecção, pois o desconhecimento da doença leva à continuidade de práticas sexuais sem prevenção. A inclusão gradual dos testes rápidos para HIV nas aldeias a partir de 2012 pode ter contribuído para a retomada no crescimento da taxa de detecção de HIV nos últimos dois anos do período de estudo, uma vez que proporcionou o aumento da cobertura diagnóstica, como observado em outros DSEI do Brasil^24^ . A predominância de casos de aids diagnosticados na faixa etária de 20 a 39 anos e de 40 a 60 anos também foi verificada no cenário nacional^b^ e em estudo conduzido entre os povos indígenas da Bolívia e do Peru, que observou um em cada dois casos entre 20 e 39 anos^19^ .

A observação das taxas de mortalidade e letalidade registradas entre os Kaiowá é relevante para a organização dos serviços de saúde, pois indica que essa etnia tem menor acesso ao tratamento antirretroviral. A diminuição das taxas de mortalidade e letalidade no Polo de Dourados, no segundo período, sugere maior estruturação dos serviços de saúde nas aldeias e articulação com os serviços de atenção especializada (SAE) para o tratamento dos indígenas com aids. O comportamento diferenciado das taxas de HIV e aids em cada um dos Polos mostra a necessidade de avaliação dos serviços de saúde separadamente, uma vez que eles se estruturam de forma diferenciada para enfrentar o problema.

As principais limitações deste estudo estão relacionadas ao uso de fontes de dados secundários, que impõe entraves à qualidade da informação. A deficiência na completude da variável raça/cor indígena no Sinan e a subnotificação de casos de HIV e aids em indígenas são fatores que influenciam no número de casos identificados, e consequentemente, na cobertura da informação.

Para ampliar a cobertura de casos de infecção pelo HIV, este estudo utilizou os registros de atendimentos diários das EMSI como fonte de dados, tendo em vista que a notificação compulsória foi incluída no Sinan somente em 2005 para gestantes^g^ e em 2014 para a população em geral^h^ . A subnotificação e incompletude dos dados da variável raça/cor dificultam a caracterização de situações de iniquidade em saúde e o estabelecimento de estratégias para o seu enfrentamento^25 , 26^ .

A despeito destas limitações, o presente estudo trouxe benefícios por abordar uma doença particularmente pouco conhecida na epidemiologia dos povos indígenas no Brasil, que traz impactos no perfil de morbimortalidade, e por se relacionar à emergência de infecções sexualmente transmissíveis e sua associação com as mudanças socioculturais resultantes do processo de interação com a sociedade não indígena.

O desafio de trabalhar as IST em um contexto interétnico implica a necessidade de apreensão de conhecimentos sobre sexualidade inerentes a cada grupo indígena^27^ e de articulação dos sistemas de saúde indígena e não indígena^28^ . O conhecimento das dificuldades logísticas, distribuição da população e áreas mais vulneráveis ajuda a definir estratégias para aumentar a cobertura diagnóstica e facilitar o acesso ao tratamento^29^ . Nesse sentido, a capacitação dos recursos humanos para trabalhar em um contexto intercultural é fundamental para aumentar a capacidade de resposta do Sasi-SUS nas ações de prevenção e tratamento das doenças.

O estudo evidenciou que o caráter endêmico do HIV e da aids, com predomínio dos casos de infecção pelo HIV na região sul do estado, pode se tornar epidêmico em alguns anos, pois já há casos em outras áreas indígenas do Mato Grosso do Sul. Entre as medidas de intervenção importantes para enfrentar o problema, destacam-se o aumento da cobertura diagnóstica, a garantia do acesso ao tratamento e as medidas de prevenção da transmissão do HIV.

## Footnotes

a UNAIDS. Prevention Gap Report. Geneva; 2016 [citado 1 jun 2017]. Disponível em http://www.unaids.org/sites/default/files/media_asset/2016-prevention-gap-report_en.pdf

b Ministério da Saúde (BR), Secretaria de Vigilância em Saúde, Departamento de IST, Aids e Hepatites Virais. Boletim epidemiológico HIV/Aids. Brasília, DF; 2017 [citado 7 maio 2018]. Disponível em: http://www.aids.gov.br/pt-br/pub/2017/boletim-epidemiologico-hivaids-2017

c Ministério da Saúde (BR), Secretaria de Políticas de Saúde, Coordenação Nacional de DST e Aids. Povos indígenas e a prevenção às DST, HIV e aids: manual de diretrizes técnicas. Brasília, DF; 2000 [citado 16 fev 2017]. Disponível em: http://bvsms.saude.gov.br/bvs/publicacoes/indigena_hiv.pdf

d Brasil. Lei 9.836, de 23 de setembro de 1999. Acrescenta dispositivos à Lei n8.080, de 19 de setembro de 1990, que “dispõe sobre as condições para a promoção, proteção e recuperação da saúde, a organização e o funcionamento dos serviços correspondentes e dá outras providências”, instituindo o Subsistema de Atenção à Saúde Indígena. Brasília, DF; 1999 [citado 10 set 2018]. Disponível em: http://www.planalto.gov.br/ccivil_03/leis/L9836.htm

e Instituto Brasileiro de Geografia e Estatística. Os indígenas no censo demográfico 2010. Brasília, DF: IBGE; 2010. [citado 16 abr 2017]. Disponível em: http://www.ibge.gov.br/indigenas/indigena_censo2010.pdf

f Ministério da Saúde (BR), Secretaria Especial de Saúde Indígena, Distrito Sanitário Especial Indígena Mato Grosso do Sul. Caracterização demográfica, étnico-cultural dos povos indígenas do DSEI Mato Grosso do Sul. Campo Grande, MS: DSEI/MS; 2014.

g Ministério da Saúde (BR), Secretaria de Vigilância em Saúde. Portaria nº 33, de 14 de julho de 2005. Inclui doenças à relação de notificação compulsória, define agravos de notificação imediata e a relação dos resultados laboratoriais que devem ser notificados pelos Laboratórios de Referência Nacional ou Regional. Brasília, DF; 2005 [citado 24 ago 2018]. Disponível em: http://bvsms.saude.gov.br/bvs/saudelegis/svs/2005/prt0033_14_07_2005.html

h Ministério da Saúde (BR). Portaria nº 1.271, de 6 de junho de 2014. Define a Lista Nacional de Notificação Compulsória de doenças, agravos e eventos de saúde pública nos serviços de saúde públicos e privados em todo o território nacional, nos termos do anexo, e dá outras providências. Brasília, DF; 2014 [citado 24 ago 2018]. Disponível em: http://bvsms.saude.gov.br/bvs/saudelegis/gm/2014/prt1271_06_06_2014.html
